# Impact of Fly Ash Fractionation on the Zeolitization Process

**DOI:** 10.3390/ma13051035

**Published:** 2020-02-25

**Authors:** Dorota Czarna-Juszkiewicz, Piotr Kunecki, Rafał Panek, Jarosław Madej, Magdalena Wdowin

**Affiliations:** 1 Mineral and Energy Economy Research Institute, Polish Academy of Sciences, Wybickiego 7A, 31-261 Kraków, Poland; dczarna@meeri.pl (D.C.-J.); pkunecki@meeri.pl (P.K.); 2 Department of Geotechnical Engineering, Civil Engineering and Architecture Faculty, Lublin University of Technology, Nadbystrzycka 40, 20-618 Lublin, Poland; r.panek@pollub.pl (R.P.); j.madej@pollub.pl (J.M.)

**Keywords:** coal combustion products, fly ash, sieving, zeolites, hydrothermal syntheses, physicochemical characterisation, zeolite X, sieving separation

## Abstract

Coal combustion product in the form of fly ash has been sieved and successfully utilised as a main substrate and a carrier of silicon and aluminium in a set of hydrothermal syntheses of zeolites. The final product was abundant in zeolite X phase (Faujasite framework). Raw fly ash as well as its derivatives, after being sieved (fractions: ≤ 63, 63–125, 125–180 and ≥ 180 µm), and the obtained zeolite materials were subjected to mineralogical characterisation using powder X-ray diffraction, energy-dispersive X-ray fluorescence, laser diffraction-based particle size analysis and scanning electron microscopy. The influence of fraction separation on the zeolitization process under hydrothermal synthesis was investigated. Analyses performed on the derived zeolite X samples revealed a meaningful impact of the given fly ash fraction on synthesis efficiency, chemistry, quality as well as physicochemical properties, while favouring a given morphological form of zeolite crystals. The obtained zeolites possess great potential for use in many areas of industry and environmental protection or engineering.

## 1. Introduction

In 2018, the global trend of increasing energy production continued, reaching +2.8% about 2017. The US and China were the main contributors to the increase in global energy production, together contributing 54% of growth in 2018. In Europe, the opposite trend was observed—energy production continued to decline owing to a slight decline in electricity production from nuclear sources, the depletion of oil and gas resources and climate policy away from coal (which for now is still a highly important, strategic energy resource for many countries) [1].

**Figure 1:**
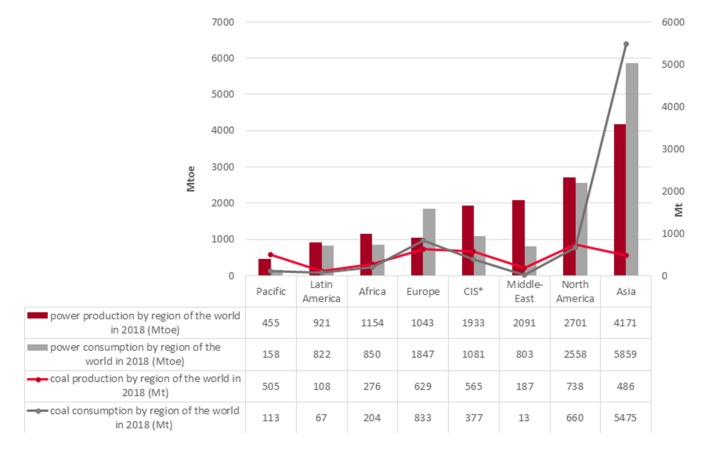
The global trend of energy and coal production/consumption in 2018 (based on [1]).

In 2018, worldwide energy consumption grew significantly by about +2.3% compared to 2017. The main reason was the high growth in electricity and gas demand, prompted by sustained economic growth and rising demand in China [1] (Figure 1).

Coal usage for energy production has fallen due to environmental protection reasons, but coal is still a strategic raw material used around the world. Despite this fact, 2018 was the third consecutive year reporting coal production growth (about +1.9% in relation to 2017). The leadership position of coal and lignite production was maintained by China (covering about 45% of global production) [1]. In 2018, coal consumption on a global scale recorded a rise of about +0.9% driven primarily by Asia (+1.8%) [1] (Figure 1).

Coal combustion in power plants and combined heat and power plants, besides being a positive aspect of energy generation, has also led to the generation of a series of gases (CO_2_, SO*_x_*, NO*_x_*, Hg^0^) and large quantities of solid waste residues such as CFA [2,3]. Among the components of fly ash are hazardous elements such as heavy metals: arsenic, lead, mercury, cadmium, chromium and selenium, as well as antimony, barium, beryllium, boron, chlorine, cobalt, manganese, molybdenum, nickel, thallium, vanadium and zinc. When those toxic elements enter the human body, they can strongly impact health and cause a several diseases such as: cancer and nervous system impacts and cognitive deficits; developmental delays and behavioural problems; heart damage; lung disease; respiratory distress; kidney disease; reproductive problems; gastrointestinal illness; birth defects; and impaired bone growth in children [2,3]. That is why attempts to facilitate the safe and sustainable utilisation of CCP are highly important in relation to energy production from coal. This article addresses this challenge regarding the utilisation of fly ash.

In recent years, many solutions for the management of fly ash have been put forward. Despite the interesting proposals for the management of ashes, the hazardous properties of fly ashes should be taken into account. The heavy metal concentration in coal fly ash varies depending on the type of coal. High levels of arsenic (2.8–6300 ppm), mercury (0.02–0.36 ppm), molybdenum (1.2–236 ppm) and selenium have been reported [4]. Several scientists have proposed the use of fly ash in agriculture, mostly as soil fertilizing additives [5,6,7,8,9], as additive in concretes [10,11,12,13,14], building materials such as bricks [15,16,17,18] and asphalts [19,20,21,22]. Utilisation via the synthesis of zeolite materials is one of the researched and considered possibilities of using fly ash [23,24,25,26,27]. The hydrothermal reaction using strong alkaline medium leads to the dissolution of many compounds from fly ash. Considering the use of zeolites as heavy metal adsorbents, knowledge about the loading of the hazardous elements and their mobility during application for wastewater treatment and soil remediation is necessary [4,28]. Studies on the mobility of metals (investigated via leaching in aqueous solutions at various pH, for example, 1, 2, 12, 13) such as arsenic, lead, chromium and cadmium in zeolites have shown that, due to the zeolitization process, heavy metal elements are immobilized in the zeolite structure, while part of the metalloid elements migrated into the wastewater. The opposite was observed in the case of fly ash, where migration of toxic elements was noticed, which confirms the need to recycle coal fly ash [29].

Zeolites are a group of solid, crystalline structures made of TO_4_ tetrahedrons called PBUs. The T atom position may be occupied by silicon or aluminium and the specific relation between Si and Al gives the zeolite structure a charge: as the aluminium content increases, the structure charge is more negative. These elements, bonded with oxygen through bridges, form a framework with chains, cavities and channels called SBUs. Inside these bigger unit systems, cations water and/or small molecules may reside. Positively charged ions can compensate for the resulting negative framework charge caused by the Si/Al ratio [30].

The majority of fly ash particles are characterised as spheroids in terms of shape and their reactivity with NaOH is different concerning the inner core of fly ash particles.

It has been observed that the smooth, outer boundary of the spherical particle, which is composed of glassy material, exhibits higher reactivity compared to quartz and mullite embedded in the inner portion of the fly ash particle. However, the amorphous silica and alumina found in the glass layer become dissolved in alkaline solutions, causing the solution to enter the inner layers. The crystalline phases of SiO_2_ present in the quartz are attacked by cation (i.e., Na^+^) as a mineralizer, leading to the formation of a soluble form of the sodium silicate and an increase in Si^4+^ in the solvent. The same applies to mullite, resulting in an increased concentration of Al^3+^ than Si^4+^ in the solvent solution [31,32].

Considering the mechanism of the zeolitization of fly ash by an alkaline solution, it is necessary to know the properties of raw fly ash in physical, chemical and mineralogical terms. According to David et al. [33] and Murayama et al. [34], differences in the size of ash particles determine their use in obtaining sorbents. It was found that CFA particle sizes ≥ 100 µm (+140 mesh), due to the high content of unburned carbon, are used to obtain sorbents such as activated carbons, while finer fractions (−140 mesh) favour the formation of zeolites.

To date, many papers have been focused on the synthesis of zeolites using raw fly ash [24,35,36] or magnetic fractions separated from it, which essentially does not participate in the zeolite synthesis reaction [37]. So far, no attempts have been made to analyse the impact of the separated fly ash fraction on the formation of zeolites in the synthesis process.

This paper offers an analysis of fly ash-derived fractions in the formation/crystallization process of zeolite syntheses. Fly ash is a carrier of silicon and aluminium which, in ash, exist mainly in the form of amorphous enamel. This form of silicon and aluminium existence allows for easy delivery of these elements to the reaction solution, in turn providing building material for zeolite frameworks. The main aim of this paper is the examination of the effect of fly ash fractions on the effectiveness of the hydrothermal synthesis of Zeolite X—known as the FAU [30]. The aim is also to indicate whether the given fractions of the starting material favour the formation of spherical, rosette-shaped or X-shaped crystals (indirect considerations on homogeneous and heterogeneous nucleation). Authors also considered whether delivering fine fractions to the solution works better (larger reaction surface with NaOH solution = more Si and Al passes into solution, homogeneous nucleation > heterogeneous nucleation) or whether larger fractions work better (providing more potential crystallization centres, heterogeneous nucleation > homogeneous nucleation) This is an innovative approach worthy of study, which has not yet been described in the specialist literature.

## 2. Experimental Details

### 2.1. Materials and Reagents

Samples of CFA (F class) were obtained from a power plant in Poland using a conventional combustion system with hard coal. Sodium hydroxide (NaOH) in the form of anhydrous pellets (>98% purity), purchased from STANLAB (Lublin, Poland), was used as an alkali activating agent.

### 2.2. Fraction Separation and Zeolite Synthesis Procedures 

Prior to hydrothermal synthesis, 1 kg of raw CFA was sieved using an electromechanical sieve shaker, whose average mesh diameters were 63, 125 and 180 µm. Fly ash was sieved for about 5 min. As a result, four fractions of fly ash were obtained: ≤ 63, 63–125, 125–180 and ≥ 180 µm.

**Figure 2:**
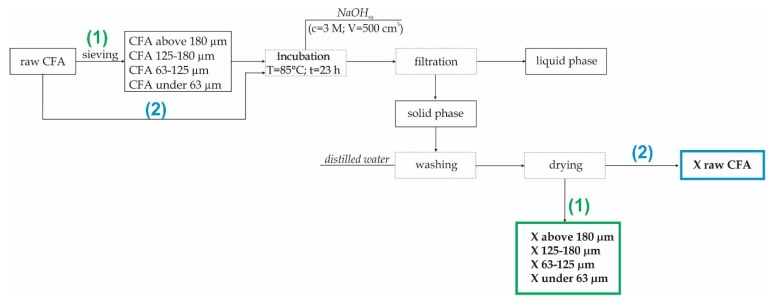
The synthesis of zeolite X from CFA (coal fly ash) preceded by a sieving process.

A total of 20 g of CFA (both CFA and separated fractions) was mixed with the alkali activating agent in a water solution (C_NaOH_ = 3 M; V_NaOH_ = 500 cm^3^). The mixture was then incubated at 85 °C for 23 h under static conditions and atmospheric pressure. After this process, the solid product was filtered and washed in distilled water several times. A flow chart for one of the samples is shown in Figure 2. All syntheses of zeolites from separated fractions (1) and raw CFA (2) (used as a comparative material) were carried out in the same manner.

### 2.3. Characterisation Methods 

The phase composition was determined via the powder XRD method using a Panalytical X’pert MPD diffractometer (with a PW 3050/60 goniometer) (Malvern, UK), a Cu lamp and a graphite monochromator. The analysis was performed within the angle range of 5–65°2θ. Panalytical X’Pert Highscore software was used to process the diffraction data. The identification of mineral phases was based on the PDF-2 release of the 2010 database, formalized by the ICDD, and on the IZA-SC Database of Zeolite Structures. The experimental calculations of the unit cell parameters were performed using UnitCell software.

Chemical analysis was performed via the EDXRF method, using the Epsilon 3 Panalytical spectrometer (Malvern, UK) with the following parameters: RTG Rh 9 W, 50 kV and 1 mA lamp. The analysis ranged from Na to Am. The sample was air-dried.

PSA was performed in order to diagnose the degree of grinding in the case of the ash samples. The study of the particle size and the distribution of the raw and milled fly ash was carried out using a laser particle size analyser, the Mastersizer 3000, manufactured by Malvern.

The morphological forms and the chemical composition of the main mineral components were determined by means of SEM with the FEI Quanta 250 FEG (Hillsboro, OR, USA).

## 3. Results and Discussion

According to [38], sieving is the simplest way to remove unburned carbon, particularly when large amounts of coarse carbon are present in raw fly ash. Many types of CFA that are rich in unburned carbon contain a relatively large amount of coarse carbon particles in fractions >150 µm. Although this often accounts for only 10% of the total mass of fly ash, these coarse carbon particles may be present in up to 50% of the total unburned coal [39].

In the case of the analysed fly ash, the highest weight percent has fractions of 63–125 µm, equating to 59.5%. On the other hand, the weight percent of the fractions with the coarsest particles (i.e., above 180 µm) is equal to 3.95% and the highest proportion of unburned coal is expected in them (Table 1).

### 3.1. Chemical Composition

Qualitative and semi-quantitative chemical composition analyses allow for a comparison of the major and minor components of raw fly ash and the fractions separated from it. Table 2 below presents the most important components in CFA and its derivatives after sieving as well as the zeolites obtained from them (the results were calculated in relation to oxides and normalised to 100%).

In the case of sieved fly ash samples, there is an insignificant trend that allows us to list two similar groups of samples. Considering the silicon and aluminium weight percent, it can be stated that samples of CFA ≤ 63 µm and CFA of 63–125 µm (smaller fractions) are characterised with a slightly higher weight percent of SiO_2_ and Al_2_O_3_ in relation to CFA of 125–180 µm and CFA ≥ 180 µm. Differences are in the range from 2.41 to 3.31 wt.% in the case of SiO_2_ and from 1.02 to 1.21 wt.% in the case of Al_2_O_3_. Thus, they may affect the effectiveness of zeolite syntheses. Mg, P and Ca are also more strongly associated with smaller fractions of fly ash. The opposite situation is observed in the case of S, K, Ti and Fe, whose content increases in samples of CFA equal to 125–180 µm as well as CFA > 180 µm. The Si/Al mass ratio of sieved ashes is similar and balanced in the range from 1.99 to 2.0. This phenomenon indicates the uneven distribution of elements constituting fly ash fractions after sieving. In the case of sieved fly ash samples, there is an insignificant trend that allows us to list two similar groups of samples. Considering the silicon and aluminium weight percent, it can be stated that samples of CFA ≤ 63 µm and CFA of 63–125 µm (smaller fractions) are characterised with a slightly higher weight percent of SiO_2_ and Al_2_O_3_ in relation to CFA 125–180 µm and CFA ≥180 µm. Differences are in the range from 2.41 to 3.31 wt.% in the case of SiO_2_ and from 1.02 to 1.21 wt.% in the case of Al_2_O_3_; thus, they may affect the effectiveness of zeolite syntheses. Mg, P and Ca are also more strongly associated with smaller fractions of fly ash. The opposite situation is observed in the case of S, K, Ti and Fe whose content increases in samples of CFA equal to 125–180 µm as well as CFA ≥ 180 µm. The Si/Al mass ratio of sieved ashes is similar and balanced in the range from 2.25 to 2.29. This phenomenon indicates the uneven distribution of elements constituting fly ash fractions after sieving. Chemical analyses of derived zeolite samples show other variabilities which may inform a deeper understanding of zeolites’ formation chemistry. Samples of zeolite X derived from raw, not sieved, fly ash are characterised by the highest weight percent of Si in relation to Al. This directly translates into the possibility of exchangeable ions being incorporated (lowest content of Na).

The weight percent of Mg and K significantly decreased in zeolite samples in comparison to fly ash fractions used involving Na. Sodium ions were delivered to the reaction system with the help of NaOH, the agent that alkalises the synthesis environment. During synthesis, Mg^2+^, K^+^ and Na^+^ ions compete for cation exchange sites in the newly created zeolite framework [30,40]. According to chemical analyses, Na^+^ displaces magnesium and potassium ions to become the dominant exchangeable cation. There is also a visible decrease in the silicon weight percent in relation to substrates. In turn, the aluminium wt.% increases in the entire set of derived zeolites samples—this leads to a reduced Si/Al ratio, which oscillates between 1.75 and 1.83. The weight percent of phosphorus falls by 300%–400% while the amount of sulphur decreases even more. This element is probably rinsed out during syntheses.

### 3.2. Phase Composition

**Figure 3:**
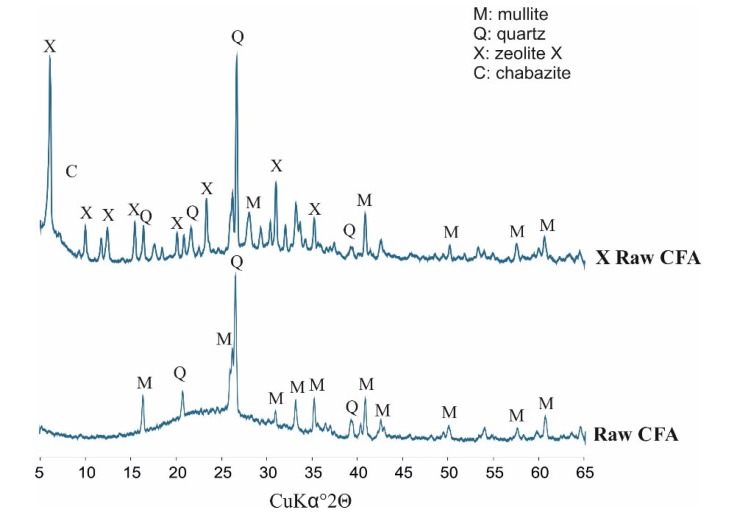
Phase analyses of raw CFA (coal fly ash).

**Figure 4:**
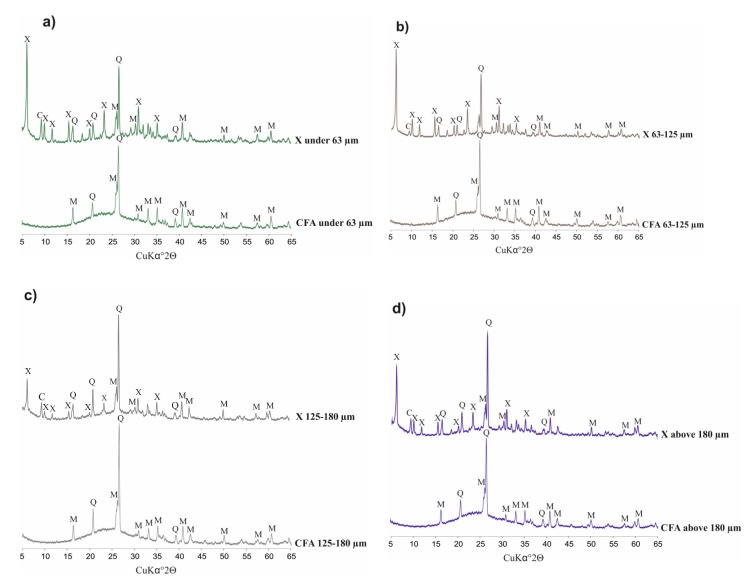
Phase analysis of CFA (coal fly ash) samples, after sieving, and zeolites obtained from them.(**a**) CFA fraction under 63 µm and X type zeolite from it; (**b**) CFA fraction from 63 µm to 125 µm and X type zeolite from it; (**c**) CFA fraction from 125 µm to 180 µm and X type zeolite from it; (**d**) CFA fraction above 180 µm and X type zeolite from it.

Figure 3 below presents diffractograms of raw CFA, while Figure 4 presents the derivatives after fraction extraction as well as the zeolites obtained from them and their residues after the synthesis processes. The whole set of CFA samples possesses quite similar mineralogical properties. Some important differences affecting the efficiency of zeolite synthesis are apparent. The following crystalline phases are present in all indicated samples: mullite (Al_6_Si_2_O_13_), represented by set of diffraction peaks with d_hkl_ values of 3.37, 3.42, 2.20, 2.54, 5.37, 1.52, 2.68, 1.44, 2.29 and 2.89 Å; and quartz (SiO_2_) represented by a set of diffraction peaks with d_hkl_ values of 3.34, 4.25, 1.81, 2.45, 2.28, 2.12, 1.54 and 1.38 Å. All of the CFA samples are characterised by a similar level of diffractogram background. This is indirect proof that the distribution of amorphous aluminosilicate in each fraction is regular. However, significant differences in peak intensity are visible. Where the characteristic quartz peaks reach the highest values (CFA of 125–180 µm) in the case of substrates, the lowest zeolite phase intensity values are achieved in the case of products (X 125–180 µm). Zeolite X phase is achieved in each synthesis and represented by set of characteristic diffraction peaks with the following d_hkl_ values: 14.47 Å (100); 8.85 Å (18); 7.54 Å (12); 5.73 Å (18); 3.80 Å (21); 2.88 Å (19). The highest degree of zeolite X crystallinity is achieved in samples of X ≤ 63 µm and X equal to 63–125 µm. This is important information from the viewpoint of the weight distribution of ash samples after the sieving process (fly ash samples in the case of X ≤ 63 µm and X of 63–125 µm possess a weight percent of 27.4 and 59.5, respectively). The visible elevation of the diffractogram background in samples after synthesis proves that there are still meaningful amounts of unreacted amorphous phase that may serve a substrate. Moreover, the characteristic peaks for quartz and mullite indicate that the dissolution process may be performed to a more effective degree. Very slight characteristic peaks of chabazite have also been observed in the entire set of diffractograms for obtained zeolites. The above results underscore the significant impact of fly ash fractions on zeolite syntheses.

According to the obtained XRD results, using UnitCell Software, some crystallographic considerations and calculations have been made, as presented in Table 3 below. An observable trend follows from them: Zeolites obtained from the finest fly ash fraction (≤ 63µm) are characterised by the least mature unit cell—the side length and the volume of the unit cell possess, in this case, the lowest values in relation to the reference zeolite on the database (reference code: 00-038-0237, sample from the PDF-2 release of the 2010 database formalised by the ICD and on the IZA-SC Database of Zeolite Structures [41]) and to the rest of the zeolites obtained from the thicker fractions. With an increase in the average grain diameter of the fly ash used, the level of maturity of the unit cell increases, in the direction where the unit cell parameters are similar to those of the reference zeolite.

### 3.3. Laser Diffraction-Based PSA

**Figure 5:**
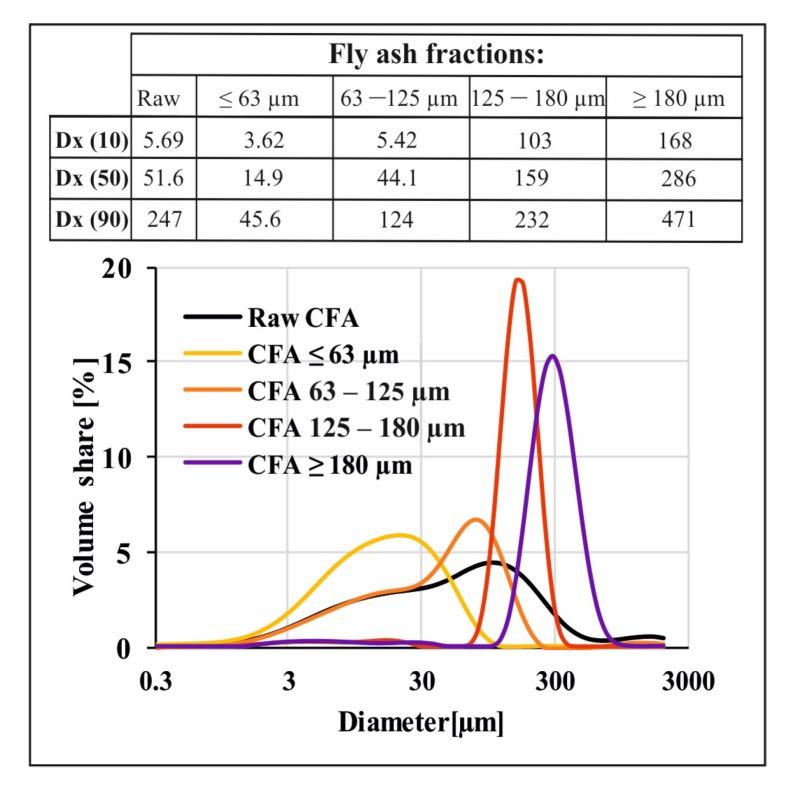
Particle size analyses of raw fly ash and fly ash fractions after sieving.

Figure 5 below presents an analysis of the particle size distribution of fly ash sieved through appropriate sieves, which confirms the progressive nature of the sieving process. The most even particle size distribution characterises the raw CFA sample. Sieved fly ash samples form two groups and have evidently different characteristics. CFA samples <63 µm and equal to 63–125 µm are slightly similar in shape to raw CFA. On the other hand, the functions of CFA samples of 125–180 and >180 µm are much sharper and more concentrated around a narrower group of particle size values. The observation of a relation with the Dx (10), Dx (50) and Dx (90) indices is more proof of the effectiveness of the sieving process but this also indirectly provides us with further knowledge about the heterogeneity of fly ash particle shapes. This is especially visible for CFA samples of 125–180 µm where the Dx (90) indicator is 232 µm. This indicates that, despite the use of a sieve with a 180 µm mesh screen, some bigger particles got through the sieve. This confirms the heterogeneous nature of the fly ash particles’ shape, which is probably needle- or spindle-like, elongated in one direction.

### 3.4. The Morphological Form Based on SEM

**Figure 6:**
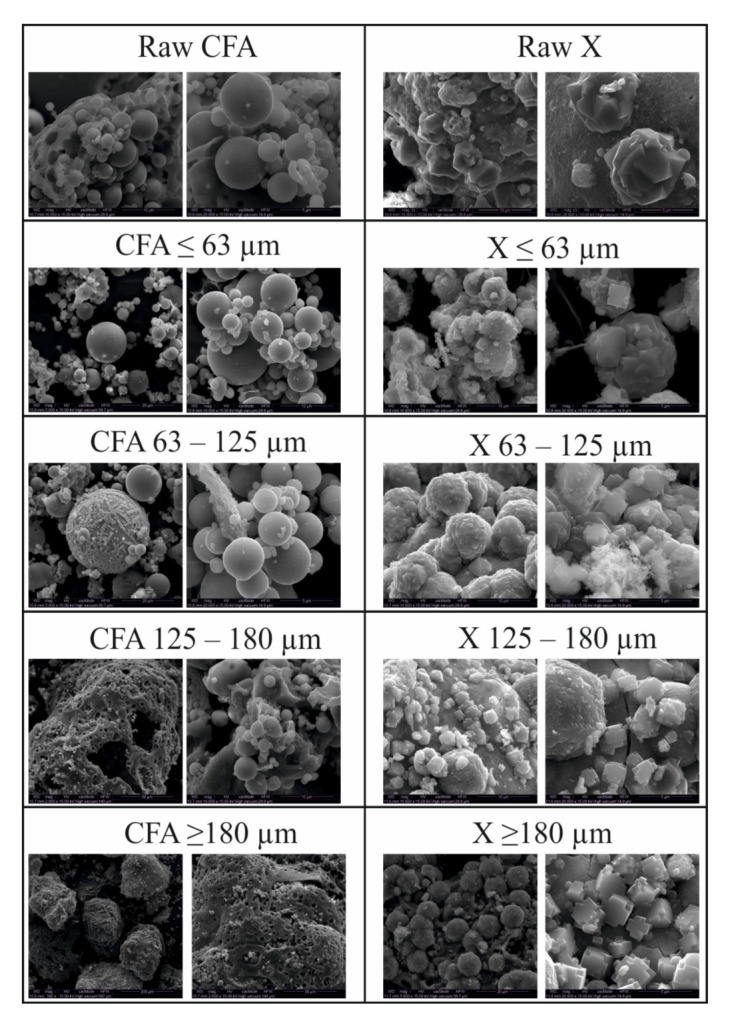
Morphological analyses of raw fly ash, fly ash fractions after sieving and zeolites obtained from them.

Analyses of SEM microphotographs (Figure 6) reveal that fly ash fractions are composed mainly of bigger aggregates of amorphous aluminosilicate enamel as well as spherical particles. Raw CFA samples ≤ 63 µm and equal to 63–125 µm are characterised by a significant share of spheres in relation to bigger aggregates. The opposite situation is observed in CFA samples equal to 125–180 and ≥ 180 µm. This is yet confirmation of the effectiveness of the sieving process. The deep insights provided by the microphotographs enable us to observe an interesting phenomenon during SEM analyses of obtained zeolite X phases. In the entire set of the obtained samples, two morphological, polymorphous forms of zeolite X crystals occurred—1) spherical, rosette-shaped and 2) X-shaped crystals. Depending on the specific fly ash fraction used as a substrate, one of the morphological forms was more abundant and dominant in a given sample. Using CFA fractions ≤ 63 µm and equal to 63–125 µm led to spherical, rosette-shaped crystallites. These are probably partly dissolved aluminosilicate spheres, on the surface of which heterogeneous nucleation occurred. In the second case, when CFA samples of 125–180 and ≥ 180 µm were used as a substrate, the X-shaped crystals of zeolite X occurred. Here, X-shaped crystals occurred individually or in clusters on the surface of partially dissolved bigger aggregates of aluminosilicate.

Rosette forms of crystals occurring in fine fractions (≤ 63 and 63–125 µm) have diameters in the range of 1–10 µm, while the X-shape crystals occurring in fractions 125–180 and ≥ 180 µm have a length in the range 1–5 µm.

## 4. Conclusions

Raw fly ash and its derivatives after sieving process were successfully used as the main carriers of silicon and aluminium in a set of hydrothermal syntheses of zeolite X, performed in a basic synthesis environment and on a laboratory scale. The results obtained during this study reveal a meaningful impact of the fly ash used on the synthesis efficiency, chemistry, quality and physicochemical properties of the derived zeolites.

According to the specialized available literature, it can be stated that great progress has already been made in increasing the efficiency of zeolite synthesis from fly ash. Several of scientists have used various techniques leading to the conversion of fly ash, nucleation and the crystallization of zeolites. One of the most important and economically effective synthesis techniques is hydrothermal synthesis. This technique was developed and described by several authors [23,24,42]. In order to increase synthesis effectiveness and the quality and quantity of zeolite as final product, a series of works has been undertaken, including the use of microwaves [43,44], ultrasonic assistance [27,45,46], the merging of fusion-hydrothermal syntheses [26,37] or molten salt methods [47]. Attempts have also been made to understand the impact of various factors affecting zeolite synthesis (e.g., temperature [48,49,50], pressure [51,52,53], Si/Al ratio [54,55,56], reaction time [25,57,58] and alkalizing agent concentration [59,60,61]). Fly ash fractions can be counted as the next important factor affecting zeolite synthesis from this CCP waste. This paper has highlighted the impact of fly ash fractions on the physicochemical properties of zeolite X phase, with particular emphasis placed on favouring a given morphological form of zeolite (X-shape or spherical, rosette) crystals, depending on the fly ash fraction. In addition, phase composition has shown that, for the finer fraction (≤ 63 and 63–125 µm) intense X zeolite peaks were observed. This is confirmed by the fact that fractions are more often used for the synthesis of zeolite X, while fractions with larger diameter are used for other types of sorbents (e.g., activated carbon due to the higher content of unburned carbon). In order to deepen our understanding of synthesis mechanisms, further research should include the use of a wider range of fly ash fractions and an attempt to synthesize other zeolite phases, such as Na-P1 (Gismondine framework), A (Linde type A framework) and Sodalite (Sodalite framework).

But taking into account the economics of the process, further investigation should also consider the necessity to separate fractions. Therefore, before considering the sieving process, specific applications of the obtained zeolite should be evaluated. Future plans aim to expand the number of fraction ranges as well as analysing the syntheses efficiency of other zeolite structures such as: Na-P1 (GIS), Na-A (LTA) and sodalite (SOD). The set of research methods will also be expanded. Additional tests are planned to show texture changes in the resulting zeolite phases (specific surface area, distribution of pores, etc.). Additionally, the Rietveld method will be used to examine the impact of individual fly ash fractions on the amount of a given zeolite phase in the final product.

## Figures and Tables

**Table 1:** Weight percent of each fraction referring to the total mass of CFA.

Fraction	CFA ≥ 180 µm	CFA 125–180 µm	CFA 63–125 µm	CFA ≤ 63 µm	Material Mass Lost
wt.%	3.95	8.25	59.5	27.4	0.9

**Table 2:** Chemical composition of fly ashes and zeolites.

		Compounds (wt.%)										
	**Fractions**	**MgO**	**Al_2_O_3_**	**SiO_2_**	**P_2_O_5_**	**SO_3_**	**K_2_O**	**CaO**	**TiO_2_**	**Fe_2_O_3_**	**Na_2_O**	**Si/Al Mass Ratio**
CFA and its derivatives after sieving	Raw CFA	1.84	26.46	56.06	0.56	0.45	4.34	3.03	1.48	7.08	-	1.87
	CFA ≥ 180 µm	1.58	23.65	53.30	0.46	0.86	4.99	2.67	1.71	10.03	-	1.99
	CFA 180–125 µm	1.66	23.34	52.86	0.46	0.66	4.96	2.91	1.69	10.66	-	2.00
	CFA 125–63 µm	1.87	24.55	56.17	0.59	0.48	4.29	2.98	1.46	6.98	-	2.02
	CFA ≤63 µm	1.79	24.36	55.27	0.64	0.48	4.52	3.30	1.58	7.35	-	2.00
Zeolites from CFA and its derivatives after sieving	X Raw CFA	2.41	24.67	50.75	0.51	0.07	3.27	4.46	1.66	8.18	3.36	1.81
	X ≥ 180 µm	2.63	26.56	48.62	0.11	0.08	1.10	2.98	1.62	9.69	6.06	1.61
	X 180–125 µm	2.22	26.81	47.94	0.10	0.12	1.42	2.63	1.58	9.08	7.56	1.58
	X 125–63 µm	2.18	26.99	47.20	0.16	0.05	1.65	3.39	1.87	8.89	6.95	1.54
	X ≤ 63 µm	2.28	27.00	47.42	0.22	0.05	1.52	3.45	1.76	8.04	7.64	1.55

**Table 3:** Unit cell parameters of obtained zeolites in comparison to the reference zeolite sample.

Unit Cell Parameters	Reference Zeolite X	X Raw CFA	X ≤ 63 µm	X 63–125 µm	X 125–180 µm	X ≥ 180 µm
a, b, c, (Å)	24.99	24.98	24.92	24.94	24.96	24.96
α, β, γ, (⁰)	90	90	90	90	90	90
V (Å^3^)	15606.26	15590.14	15490.64	15504.07	15556.50	15534.08

## Abbreviations

CCP	coal combustion product
CFA	coal fly ash
Dx	average grain diameter
EDAX	energy-dispersive X-ray analysis
EDXRF	energy-dispersive X-ray fluorescence
FAU	Faujasite framework
PBU	primary building unit
PSA	particle size analysis
SBU	secondary building unit
SEM	scanning electron microscopy
XRD	X-ray diffraction
